# Endomyocardial involvement in asymptomatic Latin American migrants with eosinophilia related to helminth infection: A pilot study

**DOI:** 10.1371/journal.pntd.0012410

**Published:** 2024-08-05

**Authors:** Abiu Sempere, Fernando Salvador, Laia Milà, Guillem Casas, Xavier Durà-Miralles, Elena Sulleiro, Rosa Vila-Olives, Pau Bosch-Nicolau, Maria Luisa Aznar, Juan Espinosa-Pereiro, Begoña Treviño, Adrián Sánchez-Montalvá, Núria Serre-Delcor, Inés Oliveira-Souto, Diana Pou, José Rodríguez-Palomares, Israel Molina

**Affiliations:** 1 International Health Unit Vall d’Hebron-Drassanes, Infectious Disease Department, Vall d’Hebron University Hospital, PROSICS Barcelona, Barcelona, Spain; 2 Centro de Investigación Biomédica en Red de Enfermedades Infecciosas (CIBERINFEC), Instituto de Salud Carlos III, Madrid, Spain; 3 Cardiology Department, Vall d’Hebron University Hospital, Barcelona, Spain; 4 European Reference Network for Rare and Low Prevalence Complex Diseases of the Heart (ERN-GUARDHEART), Amsterdam, The Netherlands; 5 Microbiology Department, Vall d’Hebron University Hospital, PROSICS Barcelona, Barcelona, Spain; 6 Departament de Medicina, Universitat Autònoma de Barcelona; Barcelona, Spain; 7 Centro de Investigación Biomédica en Red en Enfermedades Cardiovasculares (CIBERCV), Madrid, Spain; National Institutes of Health, UNITED STATES OF AMERICA

## Abstract

**Background:**

Hypereosinophilic syndrome can produce cardiac involvement and endomyocardial fibrosis, which have a poor prognosis. However, there is limited information regarding cardiac involvement among migrants from Latin America with eosinophilia related to helminthiasis.

**Methods:**

We conducted a pilot observational study where an echocardiography was performed on migrants from Latin America with both eosinophilia (>450 cells/μL) and a diagnosis of helminth infection, and on migrants from Latin America without eosinophilia or helminth infection. Microbiological techniques included a stool microscopic examination using the Ritchie’s formalin-ether technique, and a specific serology to detect *Strongyloides stercoralis* antibodies.

**Results:**

37 participants were included, 20 with eosinophilia and 17 without eosinophilia. Twenty (54.1%) were men with a mean age of 41.3 (SD 14.3) years. Helminthic infections diagnosed in the group with eosinophilia were: 17 cases of *S*. *stercoralis* infection, 1 case of hookworm infection, and 2 cases of *S*. *stercoralis* and hookworm coinfection. Among participants with eosinophilia, echocardiographic findings revealed a greater right ventricle thickness (p = 0.001) and left atrial area and volume index (p = 0.003 and p = 0.004, respectively), while showing a lower left atrial strain (p = 0.006) and E-wave deceleration time (p = 0.008). An increase was shown in both posterior and anterior mitral leaflet thickness (p = 0.0014 and p = 0.004, respectively) when compared with participants without eosinophilia.

**Conclusions:**

Migrants from Latin America with eosinophilia related to helminthic infections might present incipient echocardiographic alterations suggestive of early diastolic dysfunction, that could be related to eosinophilia-induced changes in the endomyocardium.

## Author summary

A recent study in migrants from Sub-Saharan Africa with eosinophilia related to helminth infection describes initial findings on cardiac ultrasound in the form of thickening of the posterior leaflet of the mitral valve, but with no clinical manifestations, representing the early stage of the endomyocardial fibrosis process. The aim of the present study is to evaluate the relationship between eosinophilia secondary to helminth infection and cardiac involvement in Latin American patients. In our cohort, eosinophilia in patients was mainly due to *Strongyloides stercoralis* infection. Among patients with eosinophilia, echocardiographic findings revealed a greater right ventricle thickness and left atrial area and volume index, while showing a lower left atrial strain and E-wave deceleration time when compared with patients without eosinophilia. These alterations are suggestive of early diastolic dysfunction that could be related to eosinophilia-induced changes in the endomyocardium.

## Introduction

Eosinophilia is the expansion of an eosinophilic lineage with an absolute eosinophil count (AEC) > 450 cells/μL that can be developed through three different routes: 1) primary or neoplastic, 2) secondary (helminth, drug or allergen exposure, underlying disease), and 3) idiopathic [1,2]. Hypereosinophilia is considered when AEC is greater than 1500 cells/μL, while hypereosinophilic syndromes (HES) are a group of disorders marked by the sustained overproduction of eosinophils, in which eosinophilic infiltration and mediator release cause damage to multiple organs. HES can produce a wide variety of symptoms ranging from fatigue to life-threatening endomyocardial fibrosis (EMF) and thromboembolic events which are associated with high morbidity and mortality [3,4]. Despite endomyocardial involvement being more frequent in HES of neoplastic origin [5,6], it is also associated with eosinophilia related to helminth infections diagnosed mostly in tropical and subtropical areas in Latin America, Sub-Saharan Africa and Asia, or in migrants to non-endemic countries. Although the exact physiopathology is unknown, It is suggested that proinflammatory mediators, such as eosinophil cationic proteins, may directly damage cardiac cells by affecting plasma membranes and enzyme complexes involved in the mitochondrial process [7,8].

This damage could result in regurgitation, primarily due to thickening of the posterobasal left ventricular walls, and impaired (or absent) posterior mitral leaflet motion. Furthermore, endomyocardial thickness accompanied by thrombus in the left ventricle can lead to obstruction or restriction of blood flow during filling, resulting in a restrictive filling pattern [9,10].

Research indicates that patients in Sub-Saharan Africa with helminth-induced eosinophilia exhibit cardiac alterations similar to those seen in eosinophilia from other causes, despite limited data on cardiac involvement [11,12]. A recent study describes initial findings on cardiac ultrasound in the form of thickening of the posterior leaflet of the mitral valve, but with no clinical manifestations, representing the early stage of the endomyocardial fibrosis process [13].

To the best of our knowledge, no data regarding cardiac alterations have been reported in the tropical and subtropical regions of Latin America on patients with helminth-related eosinophilia. These patients might present with different parasitic infections and carry genetic backgrounds that confer unique susceptibilities to eosinophilia. Hence, this study aims to evaluate the relationship between eosinophilia secondary to helminth infection and cardiac involvement in Latin American patients. Our goal is to enhance understanding of the risk factors and mechanisms underlying cardiac eosinophilia in this population.

## Material and methods

### Ethics statement

Procedures were performed in accordance with the ethical standards laid down in the Declaration of Helsinki as revised in 2013. The protocol for this study was approved by the Clinical Research Ethics Committee of the Vall d’Hebron University Hospital (PR-AG-357/2018). Written informed consent was obtained from all included patients.

### Patients and setting

This is a pilot observational study conducted between September 2019 and October 2021 at the International Health Unit Vall d’Hebron-Drassanes (Barcelona, Spain). Inclusion criteria were adults (> 18 years old) from Latin America, classified in two groups: participants with peripheral eosinophilia (>450 cells/μL) and a diagnosis of helminthiasis, which is considered as the presence of helminth eggs/larvae in the coproparasitological study, or positive serology for *Strongyloides stercoralis;* and participants without eosinophilia, and absence of helminth infection (negative coproparasitological study and negative serology against *S*. *stercoralis)*. Exclusion criteria were: previous anthelmintic treatment, ischemic heart disease, known structural heart disease (heart valve disease), diabetes mellitus, arterial hypertension, and Chagas disease. The included individuals underwent a transthoracic echocardiogram by a cardiologist who was blind to the presence or absence of eosinophilia. Demographic, clinical, and microbiological data were collected from electronic medical records, and entered anonymously into a database, specifically created for the study.

Because the International Health Unit Vall d’Hebron-Drassanes is a referral center for tropical diseases, recruited patients were migrants referred from various primary care centers, mostly to screen for imported diseases, or to evaluate laboratory alterations (such as eosinophilia). All patients attended to at the unit fulfilling the inclusion criteria were asked to participate in the study, and were included if they consented.

### Microbiological techniques

Stool microscopic examination was performed using the Ritchie’s formalin-ether technique. The serology technique used was the SciMedx *Strongyloides* serology microwell ELISA (SciMedx Corporation, Denville, NJ, United States). A positive serology was considered as when the optical density index was equal or greater than 2.5 [14].

### Echocardiographic study

A two-dimensional transthoracic echocardiographic (TTE) study was performed by a single experienced cardiologist certified by the European Association of Cardiovascular Imaging (EACVI), who was blinded to the patients’ status at the time of image acquisition and analysis. TTE images were acquired using a Vivid S70 (GE Healthcare, Norway), and were analyzed with the GE Echopach software v204 (GE Healthcare, Norway). Acquired TTE images included two-dimensional, M-mode, and Doppler and Tissue Doppler Imaging (TDI) in parasternal longitudinal and short axis, and apical 4, 3, and 2-chamber views, following standard protocols of acquisition. For strain analysis, only images with appropriate quality were used. Among others, the analysis variables were: left ventricular (LV) and right ventricular (RV) dimensions and systolic function (LV ejection fraction (LVEF) and TAPSE/S’, respectively), E/A waves, septal and lateral E’ waves, left atrium (LA) diameter, area, and indexed volume (LAVi), systolic pulmonary artery pressure (PAP), and thickness of valvular leaflets. Left ventricular global longitudinal strain (LV-GLS) was obtained as the average value for the 4, 3 and 2-chamber views. Right ventricle (RV) GLS was measured exclusively in the free wall, and left atrial (LA) GLS assessment was conducted in 4 and 2-chamber analysis, in accordance with previously published methods [15]. Aortic and mitral leaflet thickness were measured at the parasternal long-axis view, while tricuspid valve thickness was measured at the apical 4-chamber view. Specifically, valve thickness was measured at the body of the leaflets. Normal reference values were used to compare groups [16].

### Statistical analysis and ethical aspects

Considering the percentage of echocardiographic findings shown in a previous study (48% of the patients in the group with eosinophilia) [13]—a one-sided α value of 5%, a power of 80%, and a maximum of 15% non-evaluable subjects—we estimated the sample size at 22 assessable subjects per group: 22 participants with eosinophilia, and 22 participants without eosinophilia.

Categorical data were presented as absolute numbers and proportions, and continuous variables were expressed as means and standard deviations (when normal distribution is demonstrated using the Kolmogorov-Smirnov test), or as medians and interquartile ranges. The χ^2^ test, or Fisher exact test, when appropriate, was used to compare the distribution of categorical variables, and the Student’s *t* test or Mann-Whitney U test for continuous variables. Results with a P value of <0.05 were considered statistically significant. SPSS software for Windows (Version 19.0; SPSS Inc, Chicago, IL, USA) was used for statistical analyses.

## Results

During the study period, 51 participants were included (25 participants with eosinophilia and 26 without eosinophilia). Fourteen patients (27.4%) failed to attend their appointment for echocardiographic study; hence, 37 participants (20 with eosinophilia, and 17 without eosinophilia) were evaluated for the study. The mean age was 41.3 (SD 14.3) years, and 20 (54.1%) were men. The countries of origin of the participants were as follows: Bolivia (14, 37.8%), Ecuador (8, 21.6%), Honduras (5, 13.5%), Dominican Republic (3, 8.1%), Venezuela (3, 8.1%), Colombia (2, 5.4%), Nicaragua (1, 5.4%) and Peru (1, 5.4%). Median time of residence in Spain was 13 (IQR 3–20) years. Among the participants with eosinophilia, median eosinophil count was 1100 (IQR 1000–1850) cells/mcL, and the helminth infections diagnosed were: 17 cases of *S*. *stercoralis* infection, 1 case of hookworm infection, and 2 cases of *S*. *stercoralis* and hookworm coinfection. There were no differences regarding sex (50% of men versus 58.8%, p = 0.591) or age (mean age 44.4 years versus 37.6 years, p = 0.157) between thegroups with and without eosinophilia [1]. See Table 1 for demographic and laboratorial information of each participant.

**Table 1:** Demographic and laboratorial characteristics of the 37 included participants.

	Age (years)	Gender	Country of origin	Time of residence in Spain (years)	Eosinophil cell count (cells/mcL)	Helminth infection
Participant 1	36	Female	Nicaragua	8	0	
Participant 2	67	Male	Bolivia	22	100	
Participant 3	54	Female	Bolivia	20	300	
Participant 4	21	Male	Bolivia	17	100	
Participant 5	26	Female	Bolivia	2	0	
Participant 6	24	Female	Peru	1	1000	*Strongyloides*
Participant 7	47	Female	Dominican Republic	21	1100	*Strongyloides*
Participant 8	63	Female	Ecuador	18	900	*Strongyloides*
Participant 9	40	Male	Bolivia	16	0	
Participant 10	55	Female	Bolivia	18	200	
Participant 11	40	Female	Honduras	12	2300	Strongyloides
Participant 12	21	Female	Honduras	1	1000	*Strongyloides* + Hookworm
Participant 13	27	Male	Honduras	2	900	*Strongyloides* + Hookworm
Participant 14	40	Female	Bolivia	13	200	
Participant 15	57	Female	Ecuador	17	2600	*Strongyloides*
Participant 16	56	Male	Bolivia	12	1400	*Strongyloides*
Participant 17	46	Female	Ecuador	22	1100	*Strongyloides*
Participant 18	33	Male	Ecuador	20	1100	*Strongyloides*
Participant 19	45	Male	Ecuador	21	800	*Strongyloides*
Participant 20	32	Male	Dominican Republic	1	1000	*Strongyloides*
Participant 21	43	Male	Venezuela	1	200	
Participant 22	55	Female	Colombia	21	1200	*Strongyloides*
Participant 23	74	Female	Ecuador	13	1100	*Strongyloides*
Participant 24	51	Male	Dominican Republic	11	2000	*Strongyloides*
Participant 25	61	Male	Colombia	20	1400	*Strongyloides*
Participant 26	51	Male	Ecuador	20	900	*Strongyloides*
Participant 27	27	Male	Bolivia	3	2100	*Strongyloides*
Participant 28	45	Female	Ecuador	18	1000	*Strongyloides*
Participant 29	33	Male	Honduras	6	2300	Hookworm
Participant 30	20	Female	Bolivia	1	100	
Participant 31	22	Male	Bolivia	2	200	
Participant 32	40	Male	Honduras	3	200	
Participant 33	18	Male	Bolivia	10	100	
Participant 34	43	Female	Bolivia	18	200	
Participant 35	36	Male	Venezuela	3	200	
Participant 36	31	Male	Venezuela	4	400	
Participant 37	48	Male	Bolivia	23	200	

All patients had optimal imaging techniques, and all were in sinus rhythm. All qualitative and quantitative measures are compiled in Tables 2 and 3. When comparing the main quantitative echocardiographic findings between the two groups, participants with eosinophilia presented a significantly greater LA area (16.47 cm2 vs 14.47 cm2; p = 0.03), LA volume index (23.68 ml/m2 vs 19.29 ml/m2; p = 0.04), and a decrease in LA strain (35.47% vs 42.94%; p = 0.006) compared to those without eosinophilia. We also observed a greater right ventricle thickness (6.16 mm vs 5.25 mm; p = 0.02), and shorter E-wave deceleration time (169.65 ms vs 210.50 ms; p = 0.008). Of note, a decrease in both anterior and posterior mitral leaflet thickening was observed (p = 0.0014 and p = 0.004, respectively).

**Table 2:** Qualitative echocardiographic findings.

	Participants with eosinophilia (N = 20)	Participants without eosinophilia (N = 17)	P-value
IVS thickness (>12 mm), n (%)	1/20 (5%)	2/17 (11.76%)	0.452
LVEF (<53%)	0	0	-
GLS (>-18%)	3/15 (20%)	4/16 (25%)	0.739
RV basal diameter (>42 mm)	1/20 (5%)	2/17 (11.76%)	0.452
RV thickness (>5 mm)	15/19 (78.94%)	4/16 /25%)	0.001
TAPSE (<16 mm)	1/20 (5%)	0	0.350
S’ tricuspid (<9.5 cm/s)	0	0	-
RV strain (<20%)	5/15 (33.33%)	1/15 (6.66%)	0.068
LA volume (>20 ml)	3/20 (15%)	1/17 (5.88%)	0.373
LA volume index (>34 ml/m2)	2/20 (10%)	0	0.180
LA strain (4C) (<38%; >41%)	13/16 (81.25%)	13/16 (81.25%)	1.000
LA strain (2C) (<38%; >41%)	14/15 (93.33%)	14/17 (82.35%)	0.349
RA area (>18 cm2)	0	1/17 (5.88%)	0.272
RA volume index (>39ml/m2 in men and >33ml/m2 in women)	0	1/17 (5.88%)	0.272
IVC (>21 mm)	0	0	-
Mitral regurgitation	8/20 (40%)	9/17 (52.94%)	0.431
Aortic regurgitation	1/20 (5%)	2/17 (11.76%)	0.452
Tricuspid regurgitation	13/20 (65%)	12/17 (70.58%)	0.717
PAPs (>35 mmHg)	0	1/14 (7.14%)	0.345
Pericardial effusion	0	0	-
Ventricular diastolic dysfunction	7/20 (35%)	3/17 (17.64%)	0.236
E/A ratio (<0; >8)	1/20 (5%)	2/17 (11.76%)	0.452
Lateral E/e’ ratio (>14)	0	0	-
Septal E/e’ ratio (>14)	0	0	-
Mean E/e’ ratio (>14)	0	0	-
IVRT (<70; >100)	8/17 (47.05%)	3/17 (17.64%)	0.067
Endomyocardial infiltration	0	0	-
LV thrombus	0	0	-
RV thrombus	0	0	-
Posterior mitral leaflet thickness (>4 mm)	0	3/17 (17.64%)	0.050
Anterior mitral leaflet thickness (>4 mm)	1/20 (5%)	1/17 (5.88%)	0.906
Tricuspid valve thickness (>14 mm)	0	0	-
Aortic valve thickness (>14 mm)	0	0	-

Data are presented as number and percentage.

The specified denominator are due to limitation in performing this measurement on the entire sample.

2C: 2 chamber; 4C: 4 chamber; GLS: Global Longitudinal Strain; IVRT: Isovolumetric Relaxation Time; IVC: Inferior Vena Cava; IVS: Interventricular Septum; LA: Left Atrium; LV: Left Ventricle; LVEF: Left Ventricular Ejection Fraction; PAPs: Systolic Pulmonary Artery Pressure; RA: Right Atrium; RV: Right Ventricle; TAPSE: Tricuspid Annular Plane Systolic Excursion.

**Table 3:** Quantitative echocardiographic findings.

	Participants with eosinophilia (N = 20)	Participants without eosinophilia (N = 17)	P-value
LVEDD (mm)	45 (4.0)	44.4 (3.2)	0.632
LVESD (mm)	29.9 (4.2)	29.5 (2.4)	0.789
IVS (mm)	9.25 (1.3)	9.18 (1.9)	0.890
PP (mm)	7.95 (1.2)	7.53 (1.58)	0.371
LVEDD index (mm/m2)	25.85 (3.71)	24.72 (2.82)	0.316
LVESD index (mm/m2)	17.17 (3.24)	16.4 (1.12)	0.355
LVEDV 4C (ml)	96.95 (27.44)	105.24 (22.39)	0.327
LVESV 4C (ml)	39.3 (12.35)	41.94 (11.23)	0.504
LVEDV index 4C (ml/m2)	54.74 (12,58)	57.83 (9.66)	0.415
LVESV index 4C (ml/m2)	22.07 (5.32)	22.98 (5.15)	0.601
LVEDV 2C (ml)	98.70 (30.43)	99.65 (25.43)	0.920
LVESV 2C (ml)	39.90 (12.84)	38.88 (10.99)	0.799
Biplane LVEF (%)	59.75 (2.673)	60.88 (3.35)	0.261
LV GLS (%)	-19.58 (1.80)	-19.17 (1.30)	0.472
RV basal diameter (mm)	35.15 (5.59)	35.65 (4.40)	0.769
RV free wall thickness (mm)	6.16 (1.16)	5.25 (1.12)	0.026
TAPSE (mm)	22.60 (4.51)	21.94 (2.487)	0.595
S’ tricuspid (cm/s)	14.05 (2.12)	13.63 (1.544)	0.507
RV strain (%)	-22.66 (3.77)	-24.77 (3.76)	0.136
LA area (cm2)	16.47 (2.82)	14.47 (2.57)	0.032
LA volume (ml)	41.35 (11.74)	34.76 (9.45)	0.072
LA volume index (ml/m2)	23.68 (6.99)	19.29 (5.30)	0.041
LA strain "R" 4C (%)	36.19 (7.93)	39.69 (8.18)	0.229
LA strain "R" 2C (%)	35.47 (6.12)	42.94 (7.79)	0.006
RA area (cm2)	12.97 (2.65)	13.23 (2.99)	0.781
RA volume index (ml/m2)	16.95 (5.56)	17.31 (6.34)	0.856
IVC (mm)	11.22 (2.92)	12.63 (3.16)	0.188
PAPs (mmHg)	24.35 (4.40)	25.77 (5.31)	0.468
Mitral valve mean gradient (mmHg)	1.42 (0.37)	1.19 (0.36)	0.150
Aortic valve peak velocity (m/s)	1.30 (0.25)	1.32 (0.19)	0.841
E-wave (m/s)	0.73 (0.17)	0.79 (0.18)	0.366
E-wave deceleration time (ms)	169.65 (46.28)	210.50 (39.24)	0.008
A-wave (m/s)	0.64 (0.15)	0.62 (0.14)	0.766
E/A ratio	1.19 (0.37)	1.34 (0.54)	0.354
DTI mitral lateral ring (m/s)	0.13 (0.04)	0.13 (0.04)	0.718
DTI mitral medial ring (m/s)	0.11 (0.03)	0.12 (0.03)	0.533
Mean DTI (m/s)	0.12 (0.03)	0.13 (0.03)	0.587
Lateral E/e’	6.14 (1,89)	6.27 (2.25)	0.848
Septal E/e’	7.43 (2.55)	7.17 (2.01)	0.739
Mean E/E’	6.61 (1.95)	6.47 (1.67)	0.813
IVRT (ms)	89.59 (22.91)	82.94 (11.04)	0.289
Posterior mitral leaflet thickness (mm)	2.40 (0.59)	2.94 (0.43)	0.004
Anterior mitral leaflet thickness (mm)	2.25 (0.44)	2.76 (0.75)	0.014
Tricuspid valve thickness (mm)	2.10 (0.45)	2.29 (0.59)	0.262
Aortic valve thickness (mm)	2.15 (0.37)	2.47 (0.51)	0.034

Data are presented as mean and standard deviation.

DTI: Doppler Tissue Imaging; EDD: End-Diastolic Diameters; EDV: End-Diastolic Volume; ESD: End-Systolic Diameters; ESV: End-systolic Volume; IVC: Inferior Vena Cava; IVS: Interventricular Septum; LA: Left Atrium; LVEF: Left Ventricular Ejection Fraction; PAPs: Systolic Pulmonary Artery Pressure; PP: Pulmonary Pressure; RA: Right Atrium; RV: Right Ventricle.

Regarding the main qualitative findings, we observed a trend of a higher proportion of individuals with right ventricular thickness (78.94% vs 25%, p = 0.001), abnormal values of right ventricular free wall strain (33.33% vs 6.66%; p = 0.068), and an impaired isovolumetric relaxation time (IVRT) (47.05% vs 17.64%; p = 0.067) among patients with eosinophilia, although these differences were not statistically significant.

## Discussion

Our data revealed an increase in the area and indexed volume of the left atrium, as well as a decrease in the E-wave deceleration time and IVRT in Latin American patients with eosinophilia secondary to helminth infection. All these parameters are associated with the LV diastolic function, probably traducing an early diastolic dysfunction [17]. Interestingly, contrary to previous studies on patients with eosinophilia related to helminth infection which reported an increase in thickness [10–12], our data show a decrease in the thickness of both the anterior and posterior mitral valve leaflets. However, this finding aligns with what has been documented in cases of EMF [18]. Carranza et al. [13] reported an increase in thickness only of the posterior leaflet. However, both the geographical area (sub-Saharan Africa) and the type of helminthiasis (filariasis and/or schistosomiasis) were different from our study (of participants from Latin America mainly infected with *S*. *stercoralis*), which might be distinguishing factors.

Of note, left atrial reservoir strain was also decreased in cases compared to controls. Interest in LA strain assessment has been growing in recent years, and guidelines for the standardization of acquisition and analysis are already available [15]. It has become a promising marker of LV diastolic function, with accurate correlations with LV filling pressures and important prognostic implications, particularly in patients with preserved LVEF [19,20]. In this study, the lower LA strain observed in cases may correspond to an early or subclinical diastolic dysfunction. This could hypothetically be an initial stage of a subsequent restrictive filling pattern secondary to the eosinophilic inflammatory response. Additionally, cases presented with a greater RV thickness, which had already been reported in this pathology [18]. It is worth noting that none of the patients presented with symptoms, indicating that all these alterations are early signs of a pathology that might require additional factors to progress, such as genetic and nutritional factors, or geographical region.

EMF is the most common restrictive cardiomyopathy, and an important cause of heart failure in tropical areas of Africa, Latin America, and Southeast Asia. Its overall estimated prevalence could reach 20% of cases in endemic areas of Africa, and it has a poor prognosis [18]. Although its etiology is certainly not known, it is postulated that a combination of factors such as diet, poverty, parasitic infections, genetic predisposition, and ethnicity have been implicated in its pathogenesis [21–23]. Echocardiographic criteria for its diagnosis and assessment were defined by Mocumbi et al. in a rural area of Mozambique [18], and should meet some of the following major criteria: 1) endomyocardial plaques >2 mm in thickness or <1 mm patches affecting more than one ventricular wall; 2) obliteration of the RV or LV apex; 3) thrombi or spontaneous contrast without severe ventricular dysfunction; 4) retraction of the RV apex; 5) atrioventricular (AV)-valve dysfunction due to its adhesion to ventricular wall. Also, the minor criteria are: restrictive flow pattern across AV valves, pulmonary-valve diastolic opening, diffuse thickness of the anterior mitral leaflet, enlarged atrium with normal-size ventricle, M-movement of the interventricular septum, and flat posterior wall and enhanced density of the moderator, or other intraventricular bands.

Helminth infections have been associated with EMF [24], although they may be more strongly associated with filarial infections [25–27] than with *Schistosoma* spp [28], *S*. *stercoralis* [29], and hookworm. However, Carranza-Rodríguez et al. failed to find a correlation between cardiac involvement and any type of parasitic infection among Sub-Saharan migrants, probably due to the low number of patients included in the study [13]. Currently, echocardiographic studies on patients with eosinophilia related to parasitic infection have been primarily conducted in Africa [11–13]. This study marks the first of its kind to be performed on individuals from Latin America. Spain is a country where many migrants from Latin American countries reside, reaching numbers of 2,553,037 people [30]. One of the most frequent helminth infection-causing eosinophilia in this population is *S*. *stercoralis*, which is consistent with the findings of our study [31].

Our study has some limitations that should be acknowledged. Firstly, a total of 14 patients were lost to follow-up as they did not attend their scheduled echocardiography appointment, resulting in a reduced sample size. Additionally, the participants may not be fully representative of the general population of Latin America since most of them have been living in Spain for many years, making it likely that some helminth infections have been eliminated from the host after years of leaving the endemic area. Moreover, other nutritional and environmental factors could be involved. Some parameters could not be measured in all participants, due to technical limitations.

In summary, these data suggest that Latin American patients with eosinophilia due to helminthiasis might present incipient echocardiographic alterations suggestive of early diastolic dysfunction, which could be related to eosinophilia-induced changes in the endomyocardium. Future research should consider larger and more diverse samples to confirm our findings and further explore the relationship between eosinophilia and diastolic dysfunction.

## Supporting information

S1 Checklist STROBE Checklist. (DOC)
